# Oncological Adequacy of Laparoscopic Surgery for Bulky Gastric Cancer: Results of a Western Single-Center Series

**DOI:** 10.3390/life13122243

**Published:** 2023-11-22

**Authors:** Edoardo Maria Muttillo, Alice La Franca, Silvia Stefanelli, Alessandro Coppola, Francesco Saverio Li Causi, Rachele Anna Giannella, Elena Pino, Giorgio Castagnola, Andrea Scarinci, Genoveffa Balducci, Paolo Mercantini

**Affiliations:** 1 Department of Medical Surgical Science and Translational Medicine, Sant’Andrea Hospital, Sapienza University of Rome, 00198 Rome, Italy; edoardomaria.muttillo@uniroma1.it (E.M.M.); alice.lafranca@uniroma1.it (A.L.F.); silvia.stefanelli@uniroma1.it (S.S.); licausi.1643056@studenti.uniroma1.it (F.S.L.C.); racheleanna.giannella@uniroma1.it (R.A.G.);; 2 Department of Surgery, Sapienza University of Rome, Viale Regina Elena 291, 00161 Rome, Italy

**Keywords:** bulky gastric cancer, gastrectomy, laparoscopic, lymphadenectomy, gastric tumor, bulky tumor, D2 lymphadenectomy

## Abstract

Background: Gastric cancer is increasing worldwide and one million new cases were estimated globally in 2020. Use of the laparoscopic approach is increasing especially for subtotal gastrectomy. However, to date, solid data on locally advanced bulky tumors are lacking. The aim of this study is to assess the role of laparoscopic surgery in bulky gastric tumors. Methods: We performed an observational retrospective single-center analysis. The following data were collected and analyzed for each patient: demographics, tumor-related data, intra-operative data, peri-operative data, and pathological data. Statistical analysis was conducted, including descriptive statistics and chi-squared test, to analyze the differences between categorical variables. Results: O the 116 patients who underwent gastric surgery, 49 patients were included in the study protocol. All patients had bulky gastric tumors. Eighteen patients underwent laparoscopic gastrectomy and 31 open gastrectomy. The median number of lymph nodes removed was 28.5 (15–46) in the laparoscopic group and 23.05 (6–62) in the open group (*p* = 0.04). In total, 5.6% of patients of the laparoscopic group had <16 lymph nodes harvested and 35.5% in the open group (*p* = 0.035). No statistical differences were found between the open and laparoscopic groups in terms of surgical margins (*p* = 0.69). Conclusions: Laparoscopic surgery is still a subject of debate in locally advanced bulky gastric cancer. Limited data are available concerning Western patients. This study showed superiority in terms of the quality of lymphadenectomy and non-inferiority in terms of radical resection margins.

## 1. Introduction

One million new cases of gastric cancer (GC) were estimated globally in 2020 [1]. The most common GC histologically is represented by adenocarcinoma (approximal 90%), with the highest incidence in Southeast Asia, Central Europe, and South America. The most prevalent form of cancer in East Asia is distal cancer, accounting for approximately 80% of cases, and it is generally associated with Helicobacter pylori infection. Furthermore, there has been a recent observation of a decrease in the age group affected, with cases occurring in individuals younger than 50 years old. Pyloric-antrum gastric cancer is more commonly found in Western Europe and predominantly affects individuals with obesity and chronic gastroesophageal reflux [1,2,3]. Patients with gastric cancer typically remain asymptomatic in the early stages and therefore approximately 60% of cases are diagnosed as metastatic by the time they are identified, rendering them ineligible for curative treatment [4]. However, in recognition of the high incidence observed in South Korea and Japan, these countries have implemented widespread population screening programs, leading to improved outcomes and diagnosis at early stages. According to the guidelines of the European Society for Medical Oncology (ESMO) [1], the gold standard for diagnosis of gastric cancer is upper gastrointestinal endoscopy with biopsy, followed by a total body CT scan for lymph node staging and identification of any distant metastases. Gastric cancer can be divided into early gastric (EG), which to date is subject mainly to endoscopic treatment or—when this is not feasible—to surgical resection, and locally advanced gastric cancer (LAGC), which mostly involves peri-operative chemotherapy treatment associated with radical surgery.

The primary topics of discussion about gastric cancer within the international surgical community revolve around the extent of lymphadenectomy and the use of minimally invasive approaches such as laparoscopic or robotic surgery. Notably, laparoscopic surgery is increasingly demonstrating its advantages over open surgery, even in Western populations. In fact, in the metanalysis written by Garbarino et al. in 2022, including Western studies comparing laparoscopic versus open gastrectomy [5], the laparoscopic approach showed better results in terms of intraoperative and perioperative factors: less blood loss (WMD = −129.32 mL, *p* < 0.0001) and analgesic requirement (WMD = −1.824 days, *p* < 0.0001), faster oral intake (WMD = −1.501 days, *p* = 0.0060) and shorter hospital stay (WMD = −1.501 days, *p* < 0.0001). Regarding the short-term oncological results, LG showed lower mortality (logOR = −0.261, *p* = 0.0056) and better 3-year overall survival (logHR 0.245, *p* = 0.036). However, in this study patients both affected by early gastric cancer and locally advanced gastric cancer were analyzed, thus solid data on patients with locally advanced and bulky tumor are not provided. Thus, the debate still continues, particularly concerning the laparoscopic approach in total gastrectomy.

However, it must be stated that the data on which the guidelines are based are mainly derived from Asian cohorts that poorly represent Western reality and this constitutes a strong bias to permit strict application of Eastern guidelines to Western patients (among which higher rates of obesity, elderly age, advanced tumor stage, and the necessity of neoadjuvant chemotherapy can be found).

The extent required for lymphadenectomy is a critical point in radical gastrectomy and the current AJCC/UICC TNM recommends excision of a minimum of 15 lymph nodes for reliable staging [6]. In the East, gastrectomy with D2 lymphadenectomy is considered the gold standard for resectable gastric cancer. Concerning the findings in the Western population, in 2020 the 15-year follow up results of the Dutch Gastric Cancer Group Trial were published, and D2 lymphadenectomy was associated with a lower local recurrence (12% vs. 22% in the D1 group) and regional recurrence (13% vs. 19%). Although there was no difference in overall survival between the two groups (OS was 21% in the D1 group versus 29% in the D2 group, *p* = 0.34), gastric-cancer-related death was significantly lower in the D2 group (37% versus 48%) [7]. In the West, however, there have been no other studies evaluating the superiority of D2 over D1. In fact, the Italian gastric cancer study group (IGCSG) did not demonstrate a significant survival benefit for D2 lymph node dissection over D1 [8]. Thus, D2 lymphadenectomy is not considered a required procedure but a recommended one in the West, as extensive dissection of distant lymph nodes contributes to accurate staging of the disease. Therefore, it appears clear how the current guidelines are based on Asian data, that are not reproducible for various reasons (related to patients and malignancies) in Europe. Thus, there is no mention in the current guidelines of tumors with bulky lymph node metastasis (bulky tumors, BT), an increasing reality in the West that was considered to be an exclusion criterion in all of the major trials pertaining to the gastric cancer on which guidelines are based. Nevertheless, it is evident that large bulky tumors are becoming more prevalent in Western countries, highlighting the increasingly urgent need for data on this population. Therefore, due to the lack of scientific evidence regarding this area, the aim of this study was to compare open and laparoscopic surgery in terms of lymphadenectomy adequacy only in locally advanced bulky tumors, and considering patients coming from Western population.

## 2. Materials and Methods

### 2.1. Patient Selection

A retrospective single-center observational study was performed selecting patients diagnosed with locally advanced bulky gastric cancer undergoing total or subtotal gastrectomy at the General Surgery division at Sant’Andrea Hospital in Rome from 2016 to 2023.

Only patients ≥ 18 years old with pathological diagnosis of locally advanced gastric cancer with bulky lymph nodes were included. Patients who underwent total or subtotal gastrectomy were considered for comparison of the laparoscopic and open techniques. Exclusion criteria were age < 18 years, Siewert tumors, emergency surgery, gastrectomy for benign disease, and conversion from the laparoscopic to the open approach during surgery.

The primary outcome of the study was to compare, in a group of Western patients affected by bulky-tumor gastric cancer, the oncological adequacy of laparoscopic surgery versus open surgery in terms of surgical margins (R0) and lymphadenectomy, considered in terms of the average number of harvested lymph nodes and how many patients resulted over- or under-staged.

The secondary outcome was the evaluation of morbidity, operative time, and complication rate according to the Clavien–Dindo classification, always comparing open and laparoscopic gastrectomy.

### 2.2. Surgical Technique

**Figure 1:**
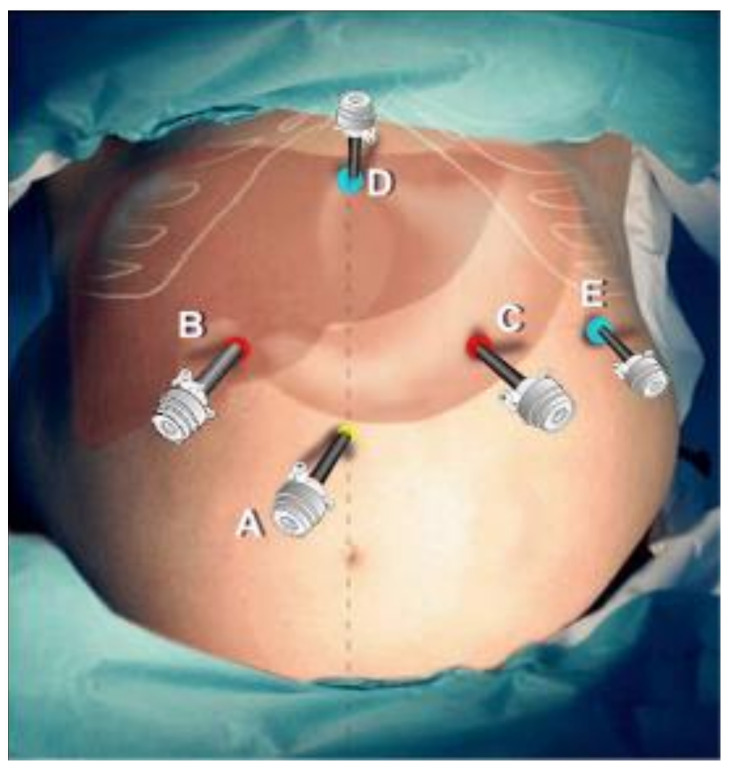
Port placement in laparoscopic gastrectomy in our center. A. A 10 mm camera port B. A 10 mm primary working port C. A 10 mm secondary working port D. A 5 mm liver retractor port E. A 5 mm assistant port.

Laparoscopic gastrectomy in our center was performed according to the standardized surgical technique, with some modifications. The patient is positioned in a supine split-leg position. Five ports are used as shown in Figure 1: a 10 mm camera port is placed along the midline in the supraumbilical region; a 10 mm primary working port is positioned along the midclavicular line in the right lumbar region; a 10 mm secondary port is placed symmetrically along the left midclavicular line; a 5 mm assistant port is positioned laterally at the left subcostal margin; a 5 mm a liver-retraction port is placed along the midline in the subxiphoid region. For total gastrectomy, the liver-retraction port is placed laterally to the right operator port in the right abdomen.

The patient is positioned in a reverse Trendelenburg to allow the transverse colon to descend. Division of the coloepiploic ligament is conducted creating an entry point to the lesser sac. The right gastroepiploic vessels are isolated and sectioned at the origin. The limphadenectomy of station 6 is performed. The right gastric vessels are isolated and sectioned. The duodenum is then sectioned using a linear stapler 1–2 cm from the pylorus. The lesser omentum is dissected and the left gastric vessels are sectioned at the origin. The abdominal esophagus is then prepared and sectioned. The limphadenectomy is conducted at the coeliac tripod, proper and common hepatic artery, and splenic artery (proximal tract). The ligament of Treitz is identified and 20–30 cm from it a loop of jejunal bowel is prepared and mobilized. An esophago-jejunal anastomosis, according to Orringer, is performed. A jejunal-jejunal side-to-side mechanical intracorporeal anastomosis is then performed at 60–70 cm.

In distal gastrectomies, the right gastric curvature is prepared and the stomach is sectioned using a intracorporeal linear stapler. A gastro-jejunal side-to-side mechanical intracorporeal anastomosis is performed, followed by a jejunal-jejunal anastomosis which is performed in the same fashion 60–70 cm distally.

Vessels are dissected using a vessel sealer and then clipped with Hem-o-lock^®^ clips (Teleflex, Morrisville, NC, USA).

**Figure 2:**
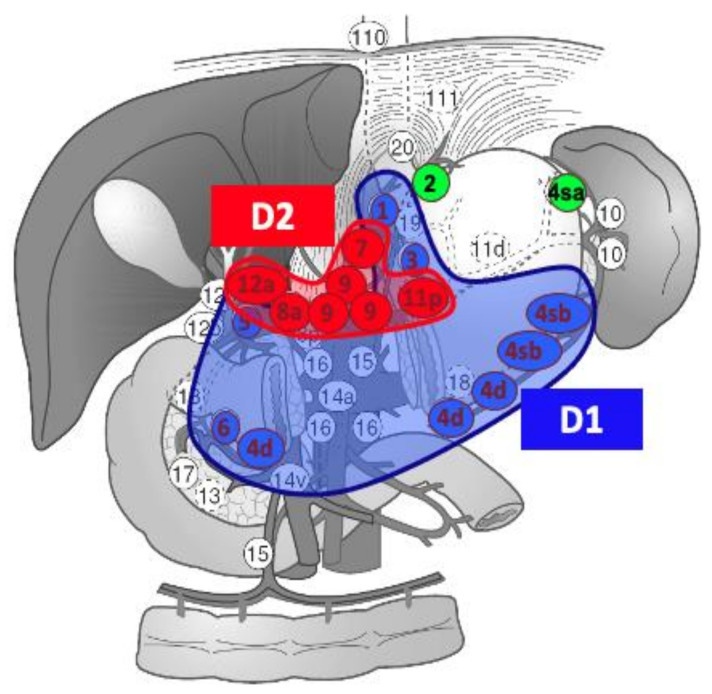
Lymphadenectomy for gastric cancer in our center. Lymph nodal stations 2 and 4sa are dissected only in a total gastrectomy.

In both distal and total gastrectomies, an indocianine green (ICG) guided modified D2 lymphadenectomy is performed. In our protocol, ICG for lymphadenectomy guidance is administered 18 h before surgery with a perilesional submucosal injection (6 mL), using a solution of 25 mg ICG reconstituted in 10 mL of aqueous solution and then diluted in 10 mL of saline solution. We perform a modified D2 lymphadenectomy. The lymph node stations are described in Figure 2.

Open gastrectomies are conducted in a similar fashion. In total gastrectomies, access to the abdominal cavity is obtained through bilateral subcostal incision. The gastroepiploic ligament is divided, accessing the lesser sac. The right gastroepiploic vessels are therefore identified and sectioned and a lymphadenectomy of station 6 is conducted. The duodenum is sectioned with a linear stapler. The lesser omentum is dissected and the left gastric vessels are sectioned. The esophagus is dissected and a circular stapler anvil is secured with a tobacco bag. The lymphadenectomy is conducted at the coeliac tripod, proper and common hepatic artery, and splenic artery (station 11p). The ligament of Treitz is identified and a jejunal loop is prepared 20–30 cm distally. A termino-lateral esophago-jejunal anastomosis is conducted with circular stapler and reinforced with manual PDS suture. The remaining cul-de-sac is sectioned with a linear stapler. A jejunal-jejunal side-to-side mechanical intracorporeal anastomosis is then performed at 60–70 cm. At this point, prophylactic cholecystectomy is conducted. Open distal gastrectomies are conducted in the same fashion as a laparoscopic procedure with a Roux-en-Y reconstruction.

ICG for lymphadenectomy guidance is not used in our center for open gastrectomies.

### 2.3. Statistical Methods

Continuous variables are presented as mean and range, while categorical variables are expressed as units and percentages. Descriptive statistics were used to summarize information relevant to the study. The differences between groups were analyzed using Student’s *t*-test for continuous variables, and Pearson’s Chi-squared test or Fisher’s exact test, as appropriate, for categorical variables. Significance was accepted at *p* < 0.05. All statistical analyses were performed using R (version 4.3.1; R Foundation for Statistical Computing, Vienna, Austria).

## 3. Results

One-hundred and sixteen patients underwent gastric surgery during the study period at our institution. Sixty-seven patients were excluded because they did not meet inclusion criteria. In detail, 10 patients had benign disease, five patients underwent emergency surgery, 47 had no lymph node involvement (N0) or did not have a bulky tumor, and data on five patients were missing.

**Figure 3:**
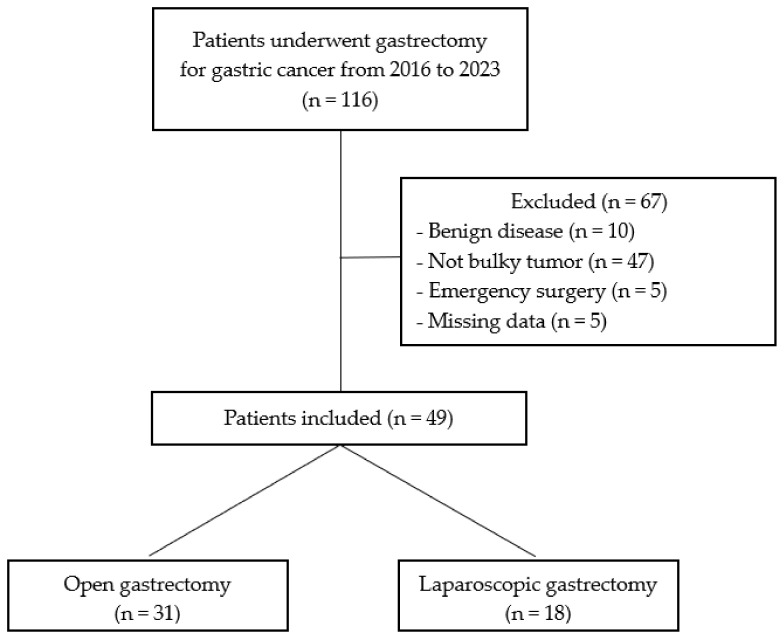
Flowchart.

The inclusion criteria were met in 49 patients: 18 of which underwent laparoscopic gastrectomy (LG, case group) and 31 open gastrectomy (OG, control group) (Figure 3).

Patients undergoing LG had a mean age of 67.2 years and an American Society of Anesthesiologist score (ASA) of 2.6; patients undergoing OG had a mean age of 62.9 years and an ASA of 2.7. Of the LG group, 27.8% were male and 72.2% were female; while in OG group 58.1% were male and 38.7% were female (*p* = 0.073) (Table 1).

The intraoperative and perioperative details of the study population are reported in Table 2. Six patients (33.3%) underwent laparoscopic total gastrectomy (LTG) and 12 (66.7%) laparoscopic subtotal gastrectomy (LSG); while eight (25.8%) patients of the 31 in the OG group received a total gastrectomy (OTG) and 23 (74.2%) a subtotal gastrectomy (OSG). Thirty-eight point nine percent of patients in the LG group and 32.3% of patients in OG group underwent neoadjuvant therapy (*p* = 0.76).

Regarding the primary outcome, the number of lymph nodes excised averaged 28.5 (15–46) in patients treated with LG, while in the controls the average number was 23.05 (6–62) (*p* = 0.04). In addition, the number of lymph nodes harvested was <16 in 5.6% of cases and 35.5% of controls, while lymph nodes harvested were >16 in 94.4% of cases and 64.5% of controls, respectively (*p* = 0.035).

Additionally, laparoscopy did not show inferiority in terms of resection radicality: rate of R0 resection was 88.9% in LG versus 80.6% in OG (*p* = 0.69).

The morbidity and complication rates, according to Clavien–Dindo classification, in the two groups (LG and OG) were not statistically significant (*p* = 0.7 and 1, respectively). Specifically, in the laparoscopic group, three patients had minor complications (CD I–II; two pneumonias and one wound infection treated with antibiotics), and one patient was reoperated on for bowel obstruction caused by an internal hernia (CD IIIb). In the open group, three patients had minor complications (CD I–II; one postoperative anemia treated with blood transfusion and two pneumonias treated with antibiotic therapy), one patient who underwent total gastrectomy had a duodenal stump dehiscence conservatively treated with percutaneous drainage (CD IIIa) and one patient was reoperated for a bowel obstruction caused by an internal hernia (CD IIIb). The total morbidity rate was 22.2% for LG and 16.12% for OG (*p* = 0.71)

Lastly, operative times were statistically significant (*p* = 0.0002) since procedures lasted 285 min for LG and 219 min for OG (Table 2).

## 4. Discussion

Over the past few years, several studies have compared the laparoscopic and open approaches for gastric cancer. However, bulky tumors were often excluded from major trials as potential bias and therefore solid data on BT remain lacking.

In 2019, the Korean Laparoendoscopic Gastrointestinal Surgery Study (KLASS 01 trial [9]) compared laparosopic subtotal gastrectomy (LSG) to open subtotal gastrectomy (OSG) including only patients with stage I gastric cancer, noting that both techniques yielded similar results in terms of 5-year overall survival. The laparoscopic approach has also demonstrated several advantages, including reduced blood loss, decreased postoperative pain, faster bowel recovery, fewer postoperative complications, and shorter hospital stays.

In 2020, the KLASS 02 trial [10] showed the non-inferiority of LSG in terms of 3-year disease-free survival-analyzed patients with locally advanced gastric cancer, but including only patients with no nodal metastasis or limited perigastric nodal metastasis, thus ruling out bulky tumors. The Chinese Laparoscopic Gastrointestinal Surgical Study (CLASS 01 trial) obtained similar results recruiting 1056 patients with clinical stage T2–T4a gastric cancer without bulky nodes [11].

Regarding laparoscopic total gastrectomy (LTG), to date there is still no consensus; the debate mainly focuses on the esophago-jejunal anastomosis, which is also the main cause of the most feared complications. In fact, even in the large LTG series, there is no consensus and standardization on which anastomosis is superior in the minimally invasive approach. However, data about LTG on patients with early-stage gastric cancer seems encouraging: in a CLASS 02 trial [12]—that included only Eastern patients with stage I gastric cancer who had undergone a total gastrectomy—morbidity and mortality rates within 30 days following surgery and intraoperative and postoperative complications did not show significant differences between LTG and open total gastrectomy (OTG). Additionally, a KLASS 03 multicenter single-arm clinical trial [13], conducted on a similar population (Eastern patients who had undergone total gastrectomy for stage I gastric adenocarcinoma) reported the safety of LTG when performed by experienced surgeons. However, as mentioned earlier, the doubt regarding esophago-jejunal anastomosis is confirmed in this study; notably, there is no one technique that is clearly superior than the other, the only data that are highlighted indicate a higher rate of stenosis for circular anastomosis (seven intracorporeal circular anastomosis vs. zero extracorporeal anastomosis) and a higher anastomosis completion time for intracorporeal (57 min intracorporeal vs. 41 min extracorporeal). Thus, as is evident from these studies, laparoscopic gastrectomy could be considered a safe alternative to open surgery, especially for early-stage gastric cancer and in the setting of extensive surgical laparoscopic expertise. However, these trials predominantly take place in the East, where the population undergoes regular screening, as opposed to the West, where tumors are often diagnosed at an advanced stage and on a population with several risk factors, such as elderly age and obesity. Consequently, as mentioned, patients in Western countries frequently present with extensive lymph node metastasis at the time of diagnosis and, besides the necessity to undergo neoadjuvant chemoterapy (another key factor that can change the general intra- and perioperative setting), the percentage of patients that undergo surgery with bulky tumors appears not negligible in the West. Despite this, there are currently no established surgical guidelines for the treatment of these patients, nor are there studies demonstrating the effectiveness of laparoscopy compared to open techniques for patients with advanced-stage gastric cancer and bulky tumors. Furthermore, minimally invasive approaches are generally not recommended by international guidelines for T4b or N2 bulky gastric cancer cases [14].

Therefore, considering the fact that Western patients, as mentioned, generally undergo surgical resection in more advanced stages due to the lack of population screening, data collected from the Eastern population cannot be superimposed on the Western population [11].

To date, there is only one recent multicenter randomized trial conducted on Western patients comparing LTG and OTG in patients with locally advanced gastric cancer previously treated with neoadjuvant chemotherapy. Ninety-six patients were included (49 OTG vs. 47 LTG) and the study showed the non-inferiority of laparoscopic surgery both in terms of radicality (median lymph node yield 43 ± 17.3 in OTG vs. 41.7 ± 16.1 LTG *p* = 0.612; R0 rate OTG 98% vs. LG 93.6%, *p* = 0.617), postoperative morbidity and one-year overall survival (90.4% in OTG vs. 85.5% in LTG, *p* = 0.701). However, both T1–T4 and N and N+ patients were included in the sample size of this study and patients with advanced nodal status (N2) represented only 14.2% of total in the open group and 8.5% in the laparoscopic group [15].

In our series of highly selected Western patients with bulky tumors, lymphadenectomy was notably more accurate in the laparoscopic group compared to the open group. This was reflected in both the mean number of harvested lymph nodes (28.05 vs. 23.05 in the open group, *p* = 0.04) and lymph node staging accuracy. Specifically, the percentage of patients with fewer than 16 harvested lymph nodes was significantly lower in the LG group (5.6%), compared to 35.5% in the OG group (*p* = 0.035).

In our opinion, several factors may explain this outcome. First of all, at our center, operating surgeons have successfully completed the learning curve for laparoscopic oncological surgery, benefiting from a solid foundation of laparoscopic expertise developed over the years, particularly in colorectal surgery. Additionally, the operating surgeons have extensive experience in open oncological gastric surgery. In fact, most of the gastrectomies in the open group selected in this study were performed during first phases of the laparoscopic gastric surgery learning curve, when patients with locally advanced tumors and bulky lymph nodes underwent open surgery more often. Therefore, increasing expertise over time has resulted in the ability to approach these difficult cases with laparoscopy.

The second reason explaining the superior quality of lymphadenectomy in the laparoscopic group is to be found in the fact that lymphadenectomy was consistently guided by ICG (Indocyanine Green). As demonstrated by Chen et al. in a randomized clinical trial published in 2020, involving 266 patients [16], ICG guidance has proven to enhance the retrieval of lymph nodes during surgery. Specifically, the study showed a significant increase in the number of retrieved lymph nodes when compared to the non-ICG group (50.5 vs. 42.0, *p* < 0.001) both in subtotal gastrectomy (49.1 vs. 39.8, *p* < 0.001), where the main results were obtained in stations 4, 6, and 7, and in total gastrectomy (52.1 vs. 42.1, *p* < 0.001), especially in stations 4sa, 7, 11d, and 12a. In our view, the most significant findings from this study pertain to the superiority of lymphadenectomy guided by ICG, which was evident in both perigastric and extra-perigastric lymph node stations.

Indeed, while there is currently no data demonstrating that a higher number of harvested lymph nodes leads to improved survival or the removal of positive lymph nodes, it is important to emphasize that accurate staging remains crucial for determining the appropriate therapeutic strategies. ICG therefore appears to be an effective affordable option, toxicity-free and easy to access and use.

It should still be noted that, in our series, there is a significant difference in terms of operating time, which should not be underestimated. Specifically, patients who underwent LG experienced an operative time that was significantly longer than those who underwent OG, with a difference of approximately one hour (285 min vs. 219 min, *p* = 0.0002), mainly attributed to the technical challenges associated with laparoscopic anastomoses, particularly the esophago-jejunal anastomosis in LTG. This issue might be partially addressed through a robotic approach, which enables enhanced wrist movements and facilitates manual sutures. Although it must be remembered that even though this longer operative time (and consequently time of anesthesia) involved patients with a higher rate of comorbidity in the LG group (obesity, elderly age, and the neoadjuvant chemotherapy treated), this did not translate into higher rate of complications in terms of morbidity rate (22.2% in LG vs. 16.1% in OG, *p* = 0.71) and Clavien–Dindo grade (CD ≥ 3 were 5.6% in LG and 6.5% in OG, *p* = 1). In conclusion, minimally invasive surgery for gastric cancer appears to hold promising oncological prospects, given its established advantages in terms of reduced blood loss, postoperative pain, accelerated recovery, and shorter postoperative hospital stays. Nevertheless, for a more comprehensive evaluation of its oncological efficacy, it will be imperative to gather robust data on laparoscopic gastrectomy performance in Western patient populations.

Standardizing ICG-guided lymphadenectomy appears essential to facilitating the design of randomized trials aimed at validating this technique in Western countries.

In the future, robotic surgery should also be considered a promising reality: Li et al. [17] conducted in 2023 a multicenter cohort study on 3552 patients in China showing a higher number of retrieved lymph nodes in the robotic group compared to the laparoscopic group—both considering all stations (32.5 vs. 40.7, *p* < 0.001) and suprapancreatic areas (13.3 vs. 11.6, *p* < 0.001)—and lower occurrence of overall complications (12.6% vs. 15.2%, *p* = 0.023) [17]. Guerrini et al. [18], in a meta-analysis published in 2020 including 17,712 patients, confirmed this result including also a Western population: the robotic group showed a higher mean number of harvested lymph nodes (mean difference 1.84, *p* = 0.0003) with a lower rate of severe complications (Clavien–Dindo ≥ 3 OR 0.66, *p* = 0.005) [18]. These results, although promising, need to be validated in the literature with large, randomized trials. Moreover, to date robotic surgery still presents some problems to be solved regarding the learning curve necessary and its availability in hospitals.

To the best of our knowledge, this study represents the first report with the aim to analysis laparoscopic surgery outcomes in a specific cohort of patients with gastric cancer characterized by bulky tumors. Nevertheless, it is essential to acknowledge several limitations in this study, including the restricted sample size resulting from the deliberate selection of bulky tumors and the retrospective nature of the investigation. Additionally, the small sample size necessitates an expansion of this work to more comprehensively reflect the Western context. Therefore, to validate the findings, it is imperative to conduct a prospective study that encompasses long-term oncological outcomes.

## 5. Conclusions

The topic of minimally invasive surgery in patients with locally advanced gastric cancer continues to be a subject of debate, primarily due to the limited data available concerning Western patients. Western patients often present with bulky tumors (excluded from all Eastern studies) and with a higher prevalence of comorbidities. In our series, consisting of Western patients with locally advanced bulky gastric cancer, laparoscopy demonstrated superiority over the open approach in terms of the adequacy of lymph node staging, characterized by a higher number of harvested lymph nodes and fewer patients being understaged. Furthermore, laparoscopy exhibited non-inferiority in terms of achieving radical resection margins (R0).

## Figures and Tables

**Table 1:** General features of study population.

Parameter		VLS (n = 18)	Open (n = 31)	*p* Value
Age, years	Mean (range)	67.2 (43–87)	62.9 (41–85)	0.32
Sex, n (%)	Male	5 (27.8%)	18 (58.1%)	0.04
	Female	13 (72.2%)	12 (38.7%)	
ASA Score	Mean (range)	2.6 (2–3)	2.7 (2–4)	0.99
Surgery, n (%)	Total Gastrectomy	6 (33.3%)	8 (25.8%)	0.74
	Subtotal Gastrectomy	12 (66.7%)	23 (74.2%)	
Multivisceral resection	Yes	6 (33.3%)	3 (9.7%)	0.058
	No	12 (66.7%)	28 (90.3%)	
Neoadjuvant chemotherapy	Yes	7 (38.9%)	10 (32.3%)	0.76
	No	11 (61.1%)	21 (67.7%)	

VLS: videolaparoscopic surgery, ASA: American Society of Anesthesiologists.

**Table 2:** Intraoperative and perioperative data.

Parameter		VLS (n = 18)	Open (n = 31)	*p* Value
Operative time, min	Mean (range)	285 (300–420)	219 (180–360)	0.0002
Morbidity, n (%)	Yes	4 (22.2%)	5 (16.1%)	0.71
	No	14 (77.8%)	26 (83.9%)	
Clavien–Dindo, n (%)	I-II	3 (16.7%)	3 (9.7%)	1
	III-IV	1 (5.6%)	2 (6.5%)	
Surgical margin, n (%)	R0	16 (88.9%)	25 (80.6%)	0.69
	R1	2 (11.1%)	6 (19.4%)	
Lymphadenectomy, n (%)	Mean (range)	28.5 (15–46)	23.05 (6–62)	0.04
	<16	1 (5.6%)	11 (35.5%)	0.035
	>16	17 (94.4%)	20 (64.5%)	

VLS: videolaparoscopic surgery.

## Data Availability

Data are contained within the article.
